# Machine learning methods to predict amyloid positivity using domain scores from cognitive tests

**DOI:** 10.1038/s41598-021-83911-9

**Published:** 2021-03-01

**Authors:** Guogen Shan, Charles Bernick, Jessica Z. K. Caldwell, Aaron Ritter

**Affiliations:** 1 Department of Epidemiology and Biostatistics, School of Public Health, University of Nevada Las Vegas, Las Vegas, NV 89154 USA; 2 Cleveland Clinic Lou Ruvo Center for Brain Health, 888 W. Bonneville Avenue, Las Vegas, NV 89106 USA

**Keywords:** Neuroscience, Neurology

## Abstract

Amyloid-$\beta$ (A$\beta$) is the target in many clinical trials for Alzheimer’s disease (AD). Preclinical AD patients are heterogeneous with regards to different backgrounds and diagnosis. Accurately predicting A$\beta$ status of participants by using machine learning (ML) models based on easily accessible data, could improve the effectiveness of AD clinical trials. We will develop optimal ML models for each subpopulation stratified by sex and disease stages using sub scores from screening neurological tests. Data from the AD Neuroimaging Initiative (ADNI) were used to build the ML models, for three groups: individuals with significant memory concern, early mild cognitive impairment (MCI), and late MCI. Data were further separated into 6 groups by disease stage (3 levels) and sex (2 categories). The outcome was defined as the A$\beta$ status confirmed by the PET imaging, and the features include demographic data, newly identified risk factors, screening tests, and the domain scores from screening tests. Monte Carlo simulation studies were used together with k-fold cross-validation technique to compute model performance metric. We also develop a new feature selection method based on the stochastic ordering to avoiding searching all possible combinations of features. Accuracy of the identified optimal model for SMC male was over 90% by using domain scores, and accuracy for LMCI female was above 86%. Domain scores can improve the ML model prediction as compared to the total scores. Accurate ML prediction models can identify the proper population for AD clinical trials.

## Introduction

The global impact of Alzheimer’s disease (AD) is immense^1^. With worldwide rates expected to triple in the next decade, the development of successful strategies to combat AD has become a global health imperative^2^. One such strategy is the identification of individuals with AD prior to the onset of dementia^3^. Alzheimer’s disease is now conceptualized as a continuous disease with a long asymptomatic phase in which neuropathological substrate accumulates eventually leading to stages of mild cognitive impairment (MCI) and finally to overt functional decline and dementia. Early diagnosis of AD has been associated with a variety of benefits including increased survival time^4^, improved psychological well-being for patients and their families^5^, and lower health care costs^6,7^. Perhaps most compelling is emerging data from clinical trials of disease modifying therapies (DMT) clearly demonstrating that meaningful therapeutic success will likely require from early intervention^8^.

Amyloid-$\beta$ (A$\beta$) is one of the two hallmark pathologies for diagnosis of AD^9^. AD is characterized by a long preclinical stage which is referred to be as mild cognitive impairment (MCI). A$\beta$ has been the target of disease modified therapies (DMTs) in many AD clinical trials. One of the most recent drugs is aducanumab which is believed to be able to reduce deposits of A$\beta$. The results from its Phase 3 study indicated a statistically significant reduction after 78 weeks in the primary outcome: Clinical Dementia Rating-Sum of Boxes (CDR-SB) score, in the high-dose aducanumab group as compared to the placebo group. This A$\beta$ targeted DMT could be the first new AD treatment in nearly two decades. A$\beta$ status can be confirmed by using either cerebrospinal fluid (CSF) or positron emission tomography (PET) imaging. CSF is invasive and potentially painful for patients, and sometimes a participant can’t have a lumbar puncture because of a back deformity, infection, or possible brain herniation. Amyloid PET imaging is preferable in certain scenarios, but its utilization in clinical and trial settings is limited due to patients’ concerns (e.g., radiation), and high costs which are often not covered by insurances. Thus, developing tools to accurately predict the A$\beta$ status offers an attractive approach^10,11^.

One potential solution for overcoming this problem is to utilize affordable global screening tools that can be rapidly and inexpensively administered to diverse populations. The Montreal Cognitive Assessment (MoCA) is one such candidate^12^. Now in widespread use, the MoCA is a brief screening tool that takes approximately 10 min to administer and score. It includes 12 individual tasks grouped into seven cognitive domains (visuospatial/executive; naming; memory; attention; language; abstraction; and orientation). Scores on each task are summed to yield a total score, with maximum total of 30. Using a cutoff of 26, quantitative analysis of the MoCA shows that it has good sensitivity and specificity for individuals with dementia but variable specificity in MCI stages (76% with a range of 19–98%)^13^. Because the narrow range of scores within each cognitive domain limit traditional statistically inquiry, less research has been conducted to indicate whether qualitative analysis (domain-level performance) improves its diagnostic accuracy^14,15^.

MCI and AD patients are characterized by heterogeneity in sex and APOE $\varepsilon 4$ status^16^. Women with AD decline more rapidly in cognition than men with AD from longitudinal studies including the ADNI^17–21^. In a study to investigate the longitudinal change in ADAS-Cog in MCI patients using the ADNI data, cognitive decline is greater in females than males, and APOE $\varepsilon 4$ carriers have a significant effect on both slope and curvature of ADAS-cog change as compared to non-carriers^22^. Women have better verbal memory than men on average, across the lifespan. In the context of AD, this memory advantage appears to persist in women with normal cognition, despite presence of measurable pathological changes, including presence of brain beta-amyloid^10,15,19,23–25^. This advantage has also been suggested to result in memory measures being less effective in screening women versus men for early AD-related changes^26,27^.

Machine learning (ML) methods hold promise for improving diagnostic classification above current processes and have been successfully applied to studies of individuals with early AD. However, current approaches utilized only a very few ML methods based on commonly used measures, and the k-fold cross-validation resampling procedure was traditionally used to evaluate model performance^11,28–30^, but the results are not reliable with only one simulation. We will use Monte Carlo simulations along with the k-fold cross-validation technique to provide reliable comparisons between the considered ML models^31–33^. The primary purpose of the current paper is to explore whether incorporating domain level scoring on the MoCA, in combination with several other widely available screening tests such as the Alzheimer’s Disease Cognitive Assessment (ADAS-Cog) and the Mini-Mental Status Examination (MMSE) into a novel machine learning (ML) algorithm improves diagnostic classification of AD in early stage individuals. It was hypothesized that incorporation of domain level scoring of these screening tests would improve performance above using the total scores in the ML models for each subpopulation.

## Methods

### Study designs and participants

Data used in this project were obtained from the ADNI database in June 2020 (http://adni.loni.usc.edu/)^34,35^. The ADNI is an ongoing longitudinal cohort of early stage AD research participants that has enrolled more than 1800 participants since 2004. Although a continuous study, there have been several phases of ADNI: ADNI-1, ADNI-Go, ADNI-2, and ADNI-3 (current). For our analysis, we wanted to select research participants at the earliest stages of symptomatic disease. This required us to select participants from different ADNI studies. Individuals with significant memory concern (SMC) were selected from ADNI-2, and ADNI-3 because the SMC cohort was added in the ADNI starting from ADNI-2 to address the gap between healthy controls and MCI. Individuals with early MCI (EMCI) or late MCI (LMCI) were selected from ADNI-GO, ADNI-2, and ADNI-3. Because ADNI-1 used Pittsburgh Compound-B (PIB) to determine amyloid positivity, we did not use data from ADNI-1, but LMCI participants initially enrolled in ADNI-1 were included if they had follow up visits in the following three phases. In the ADNI study an individual’s diagnosis is rendered based on current clinical criteria used in conjunction with performance on psychometric testing. SMC is defined by having a significant memory concern but no impairment on the Logical Memroy II subscale (Delayed Paragraph Recall, Paragraph A only) from the Wechsler Memory Scale-Revised, while EMCI and LMCI are the two complementary groups of mild cognitive impairment (MCI), and are distinguished by performance on the Logical Memroy II subscale^36^.

Amyloid positivity was determined quantitatively. We computed the standardized uptake value ratio (SUVR): the average of weighted cortical retention means divided by the whole cerebellum SUVR, where frontal, cingulate, parietal, and temporal regions were used in the calculation of cortical retention means with a threshold of 1.11 used to define the binary amyloid status^35,37,38^.

In ADNI, participants are assessed at regular visits. These assessments are used to render a diagnosis. As a result, an individual’s diagnosis may change during the course of the study. For our study we analyzed data collected from the baseline visit as this is the visit when amyloid positron emission tomography (PET) occurs. The sample size and characteristics of individuals used in our analysis are presented in Table 2. To account for the important moderating factors of sex we further stratified each subgroup by sex.

The three diagnosis groups (SMC, EMCI, and LMCI) were defined by their baseline diagnostic results. Paticipants’ amyloid status were obtained from the baseline visit, or the nearest visit having the amyloid status outcome and having the same diagnosis as baseline when the amyloid status was not available at baseline. We used that visit date to merge with other data files (e.g., cognitive measures). Sex is an important moderation factor in AD research^19,20^. For each diagnosis group, we stratified data into two subgroups by sex: Female or Male. The sample sizes for each subgroup were presented in Table 2.

### Model creation

#### Demographics

To build our model we attempted to incorporate known risk factors for amyloid positivity. Five demographic data were obtained from the ADNI: age, race (White, African American, or others), years of eduction, Hispanic ethnicity, and marital status (married, never married, divorced, or widowed). Due to small percentages of participants other than White or African American, we combined them as one group. APOE $\varepsilon 4$ was one of the three strong risk factors for amyloid status prediction in addition to age and ADAS-cog^39^. Family history of dementia^40^, history of hypertension^41^, and the Geriatric Depression Scale (GDS-15) scores were included in the ML models.

#### Sex

Women and men differ significantly in terms of neuropsychological test performance, disease trajectory, and interaction with APOE $\varepsilon 4$ status. Women’s advantage in verbal memory has been suggested to result in memory measures being less effective in screening women for early AD changes. Similarly, given that women’s strong memory might have a masking effect early in the disease process, predicting presence of brain amyloid with memory test scores is expected to be less effective for women, particularly for women with no detectable memory deficits. Based on the finding of the heterogeneity in sex and APOE $\varepsilon 4$ status in MCI and AD, it is critical to build separate statistical prediction models for each subpopulation stratified by sex and APOE $\varepsilon 4$ status. Based on these differences we built separate models for men and women at each disease stage.

#### Cognitive tests

The neuropsychological scores from the following four tests were included as features in the machine learning models: (1) Clinical Dementia Rating-Sum of Boxes (CDR-SB), (2) Mini Mental State Exam (MMSE), (3) Montreal Cognitive Assessment (MoCA), and (4) the 13-item ADAS-cog. For the MoCA score and the ADAS-cog score, we also included their domain level scores. Standard administration of the MoCA consists of 12 individual tasks grouped into seven cognitive domains: (M1) visuospatial/executive, (M2) naming, (M3) attention, (M4) language, (M5) abstraction, (M6) memory, and (M7) orientation. The ADAS-cog-13 include 13 domain areas: (A1) Word Recall, (A2) Commands, (A3) Constructional Praxis, (A4) Delayed recall, (A5) Naming, (A6) Ideational Praxis, (A7) Orientation, (A8) Word Recognition, (A9) Recall instructions, (A10) Spoken language, (A11) Word finding, (A12) Comprehension, and (A13) Number cancellation^42^.

The narrow range of scores within each domain (range from 0 to 12), makes application of traditional statistical methods to domain-specific performance difficult. As a result, the predominance of MoCA-related research has focused on total scores and likely underestimates the full utility it may provide as a screening tool. Domain level scores provide in essence, a “mini-cognitive profile” that may provide a more granular view of an individual’s cognition.

### Machine learning models

We built ML models with by using both Monte-Carlo simulations and ten-fold cross-validation procedure. In each simulation, the complete data were split into a training data set (80%) and a testing data set (20%), where the training data set will be used in ten-fold cross-validation to build the prediction model, and the testing data set will be used for validation and calculating model performance metrics.

ML models can be used to improve amyloid positivity prediction by using the easily accessible data. We applied widely used ML methods to build an optimal model with the highest average accuracy from 1000 simulations. Due to variation in splitting data into a training data set and a testing data set, a few simulations are not sufficient enough to provide reliable results. Thus, we run the simulation for 1000 times to identify the optimal ML model with reliable conclusions.

#### ML methods

We built ML predictive models with the statistical package *caret* in R^43,44^, using the following supervised ML methods: linear discriminant analysis (LDA), k-nearest neighbor (kNN), Decision trees (DT), support vector machines (SVM) and random forests (RF). The LDA classifier finds a linear combination of features that characterizes or separates two or more classes. SVM finds a decision function that maximizes the margin around the separating hyperplane by modeling a mapping from features to labels as a combination of kernels. In Table 1, we list the 15 ML models along with the method values used in the R function.

**Table 1:** The 15 ML models from the R package *caret*.

Model ID	ML model	Method value in R
1	Linear discriminant analysis	lda
2	Factor-based linear discriminant analysis	RFlda
3	Generalized linear model	glm
4	Random forest	ranger
5	Recursive partitioning and regression trees	rpart
6	k-nearest neighbors	knn
7	Support vector machines with radial basis function kernel	svmRadial
8	Support vector machines with linear kernel	svmLinear
9	Support vector machines with polynomial kernel	svmPoly
10	Random forest	rf
11	Stochastic gradient boosting	gbm
12	Boosted logistic regression	LogitBoost
13	tree models or rule-based models	C5.0
14	Bagged CART	treebag
15	Boosted classification trees	ada

#### Performance metrics

The optimal ML model is identified as the one having the highest average accuracy. Accuracy is commonly used to assess the performance of a ML model: the proportion of all classes that are correctly predicted^10,45^ which is defined as:

$$\begin{aligned} Accuracy=\frac{TP+TN}{TP+TN+FP+FN}, \end{aligned}$$

where TP, FN, TN, and FP are the numbers of true positive, false negative, true negative, and false positive, respectively. It is easy to show that the total sample size is $N$ = TP+TN+FP+FN, and $N^{+}$ = TP+FN and $N^{-}$ = TN+FP are the number of participants with positive and negative amyloid, respectively.

The Matthews Correlation Coefficient (MCC) can be considered as an alternative of accuracy to assess the model performance. The MCC is equivalent to the Pearson correlation coefficient between actual and predicted amyloid status, with the range from − 1 (perfect misclassification) to 1 (perfect classification)^46,47^. The MCC is defined as

$$\begin{aligned} MCC=\frac{TP\times TN-FP\times FN}{\sqrt{(TP+FN)(TP+FP)(TN+FP)(TN+FN)}}. \end{aligned}$$

The MCC is a reliable statistical measure, and it has a high score only if the prediction obtained good results in all of the four confusion matrix categories (high values of TP and TN, and low values of FN and FP)^48^. The Other performance metrics were also calculated and compared: sensitivity, specificity, positive predictive value (PPV), and negative predictive value (NPV).

#### Feature selections

For a study with a total of *F* features, the total number of all possible feature combinations is $2^F$. It increases exponentially as *F* goes up. For a study with 20 features, the number of all possible combinations is over 1 million. It is not computationally feasible to search over all possible combinations to identify the optimal feature set for each ML method. To reduce the computational intensity, we propose using a stochastic ordering approach in conjunction with the forward model selection approach^49^. The stochastic ordering approach is traditionally used in exact statistical inference to order the sample space which is sorted by a test statistic including point estimates and confidence limits^11,47,49,50^.

We used the forward model selection approach with the Akaike Information Criterion (AIC) as the criteria to determine the ordering of these features. The first step is to fit *F* models with one of the *F* features in each model. The model with the smallest AIC is selected and its associated feature is assigned as the feature in the first place, denoted as $X_{(1)}$. In the second step, we fit $F-1$ models with one of the remaining features after the first step, and $X_{(1)}$ in the model. The second feature is the one from the model with the smallest AIC among these $F-1$ models. Suppose the second feature is $X_{(2)}$. Following this procedure, the following ordered $F-2$ features are determined: $X_{(3)}, \ldots , X_{(F)}$. In this article, a multiple logistic regression model is the statistical model used to determine the feature ordering.

Instead of $2^F$ feature combinations, we used the *F* combinations: $Z_i=\{X_{(1)},\ldots ,X_{(i)}\}$, where $i=1, 2, \ldots ,$ and *F*. The new stochastic ordering provides an efficient way to determine the importance of these features to predict amyloid positivity, and it provides an efficient route to search for the optimal set of features.

## Results

We built ML models for female and male within each diagnosis group, with a total of 6 subgroups: SMC female, SMC male, EMCI female, EMCI male, LMCI female, and LMCI male. Table 2 presents the demographics and clinical characteristics of these 6 subgroups. The rate of amyloid positivity in female was close to that in men in the EMCI group and the LMCI group, while female had a much higher rate than male in the SMC group. Male were generally older and tended to have a higher level of education than female in each group. The ADAS-Cog-13 appeared to increase as disease was progressed, and females had better performances than men in general. We also present the pvalue in comparing the three groups (SMC, EMCI, and LMCI) for each characteristic. The following characteristics are not significant in comparing the three groups: hispanic ethnicity, race, marital status, family history of dementia, and history of hypertension. The remaining demographics and clinical characteristics show statistical differences between the three groups.

**Table 2:** Characteristics of the 6 subgroups from the ADNI.

	SMC			EMCI			LMCI			pvalue
	F	M	pvalue	F	M	pvalue	F	M	pvalue	
N	100	70		141	176		102	134		
Amyloid (%)	41 (41.0)	19 (27.1)	0.063	65 (46.1)	89 (50.6)	0.429	69 (67.6)	87 (64.9)	0.662	< 0.0001
Age	70.9 (5.9)	73.4 (6.1)	0.010	70.6 (7.8)	72.4 (7.0)	0.035	72.6 (8.5)	75.7 (8.3)	0.005	< 0.0001
Edu	16.5 (2.7)	17.3 (2.2)	0.044	15.6 (2.7)	16.5 (2.6)	0.003	15.7 (2.6)	16.5 (2.8)	0.015	0.0075
Hisp (%)	5 (5.0)	1 (1.4)	0.214	8 (5.7)	7(4.0)	0.480	4 (3.9)	1 (0.7)	0.093	0.3653
Race			0.279			0.607			0.035	0.0662
Whites	87 (87.0)	66 (94.3)		129 (91.5)	166 (94.3)		93 (91.2)	131 (97.8)		
African American	8 (8.0)	2 (2.9)		4 (2.8)	3 (1.7)		7 (6.9)	1 (0.7)		
Other	5 (5.0)	2 (2.9)		8 (5.7)	7 (4.0)		2 (2.0)	2 (1.5)		
Marry status			0.079			$<0.01$			$<0.01$	0.5913
Married	67 (67.0)	56 (80.0)		89 (63.1)	156 (88.6)		59 (57.8)	116 (86.6)		
Never married	9 (9.0)	2 (2.9)		9 (6.4)	5 (2.8)		4 (3.9)	1 (0.7)		
Divorced	9 (9.0)	8 (11.4)		25 (17.7)	11 (6.2)		14 (13.7)	9 (6.7)		
Widowed	15 (15.0)	4 (5.7)		18 (12.8)	4 (2.3)		25 (24.5)	8 (6.0)		
APOE $\varepsilon 4$			0.176			0.122			0.485	0.0001
0 copy	59 (59.0)	50 (71.4)		89 (63.1)	91 (51.7)		45 (44.1)	69 (51.5)		
1 copy	36 (36.0)	19 (27.1)		43 (30.5)	69 (39.2)		44 (43.1)	48 (35.8)		
2 copies	5 (5.0)	1 (1.4)		9 (6.4)	16 (9.1)		13 (12.7)	17 (12.7)		
ADAS-13	9.4 (4.3)	11.7 (4.5)	0.001	12.2 (5.7)	13.5 (5.4)	0.045	18.0 (8.2)	18.6 (7.0)	0.573	< 0.0001
MoCA	26.2 (2.6)	25.2 (2.7)	0.017	24.1 (3.1)	23.7 (2.9)	0.252	22.0 (3.8)	22.3 (3.1)	0.659	< 0.0001
CDRSB	0.1 (0.4)	0.1 (0.3)	0.193	1.2 (0.8)	1.3 (0.8)	0.180	1.7 (1.0)	1.7 (1.2)	0.931	< 0.0001
MMSE	29.1 (1.2)	29.0 (1.2)	0.884	28.4 (1.7)	28.2 (1.6)	0.162	27.4 (2.0)	27.5 (1.8)	0.816	< 0.0001
Family hist (%)	67 (67.0)	42 (60.0)	0.349	95 (67.4)	109 (61.9)	0.315	62 (60.8)	75 (56.0)	0.458	0.1776
Hypertension (%)	41 (41.0)	33 (47.1)	0.427	64 (45.4)	97 (55.1)	0.085	43 (42.2)	66 (49.3)	0.279	0.7189
GDS	1.0 (1.0)	0.9 (0.9)	0.625	2.0 (1.6)	1.6 (1.5)	0.019	2.2 (1.9)	1.8 (1.6)	0.137	< 0.0001

The pvalues in the last column are for the difference between the three groups (SMC, EMCI, and LMCI).

For ML models using total scores, the 13 features in Table 2 were all included in the model. For ML models using domain scores, the 7 domain scores from MoCA and the 13 domain scores from the ADAS-cog-13 were included as features in addition to the 13 features in ML models using total scores. For a categorical feature (e.g., Hispanic ethnicity), it is possible that almost all the participants belong to one category which could cause the failure of the ML model building. If that dominate category (e.g., non-Hispanic) had the participants more than the sample size in that subgroup minus 5, that feature was removed from the features in building ML models for that subgroup.

**Figure 1:**
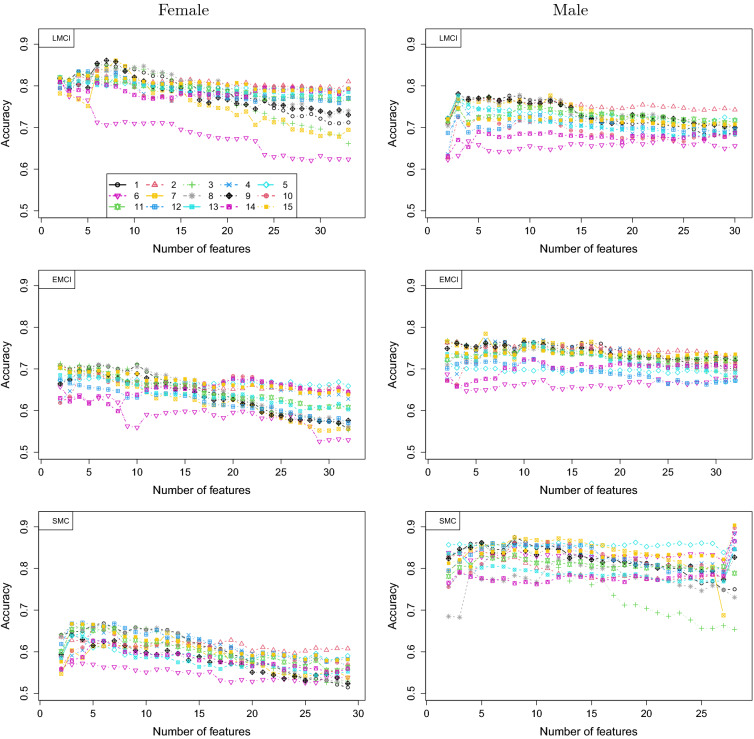
Accuracy of the 15 ML methods using the domain scores for the 6 subgroups stratified by diagnosis and sex.

We utilized the proposed stochastic ordering method for the feature importance ordering in each subgroup. It should be noted that the ordering of features in each subgroup could be different because the importance of features in predicting amyloid positivity varies in each subgroup. Figure 1 shows the accuracy of each ML method using domain scores, as a function of the numbers of features in each subgroup. The accuracy lines are quite smooth under the new stochastic ordering feature selection method. It can be seen that accuracy for male is much higher that that for female within SMC or EMCI, while it is reversed in LMCI with a higher accuracy for female. The highest accuracy is often achieved with less than half of the features, except the case for SMC male.

We presented the optimal ML method using domain scores and the associated number of features for each subgroup in Table 3. It should be noted that the stochastic ordering of features in each subgroup is often different from each other. The optimal numbers of features are often small, except the case for the SMC male group. The SVM methods were the best in half of the cases: the SVM with polynominal kernel (*svmPoly*) for both subgroups in the LMCI, and the SVM with radial kernel (*svmRadial*) for EMCI male. The other 3 optimal methods were: a generalized linear model (*glm*), a boosted Logistic Regression (*LogitBoost*), and a boosted classification trees method (*ada*).

**Table 3:** The optimal ML model for each subgroup.

Diagnosis	Sex	Optimal ML method	Num	Features
LMCI	F	SVM with polynomial kernel	7	APOE $\varepsilon 4$, ADAS total, ADAS-Q2, 4, 9, 12, Family Hist, GDS
LMCI	M	SVM with polynomial kernel	3	APOE $\varepsilon 4$, ADAS total, ADAS-Q2,
EMCI	F	Generalized linear model	2	Age, APOE $\varepsilon 4$
EMCI	M	SVM with radial kernel	6	Age, APOE $\varepsilon 4$, marry status, ADAS-Q4, Q5, Q6
SMC	F	Boosted logistic regression	4	APOE $\varepsilon 4$, MoCA total, ADAS-Q8, ADAS-Q11
SMC	M	Boosted classification trees	27	All features

We compared accuracy of optimal ML models using domain scores and total scores in Table 4. The ML models using domain scores had substantial accuracy gain as compared to those based on total scores in the following three subgroups: LMCI female (3.4% increase), LMCI male (3.1% increase), and SMC male (4%). In the EMCI groups, the optimal ML models using domain scores were similar to those using total scores. In addition to accuracy, we presented the other five ML model performance matrix (MCC, sen, spe, PPV, and NPV) in Table 4 for the identified optimal ML models. When the overall accuracy was similar between female and male (e.g, the EMCI group), all other model performance matrix were similar as well. When the accuracy of the optimal ML models using domain scores was higher, the MCC was higher and other performance measures (sen, spe, PPV, NPV) were better balanced (e.g., sen and spe were close to each other).

**Table 4:** Comparing ML models using domain scores or total scores based on 1000 simulations.

Diagnosis	Sex	Scores	Accuracy (%)	MCC	Sen (%)	Spe (%)	PPV (%)	NPV (%)
LMCI	F	Domain scores	86.1	0.694	88.8	80.4	91.2	78.8
LMCI		Total scores	82.7	0.668	78.6	91.7	95.6	67.9
LMCI	M	Domain scores	78.1	0.534	80.9	72.7	85.2	68.0
LMCI		Total scores	75.0	0.424	88.2	50.0	76.8	70.2
EMCI	F	Domain scores	71.2	0.428	66.3	75.5	71.0	72.8
EMCI		Total scores	71.3	0.430	66.7	75.3	70.9	73.1
EMCI	M	Domain scores	78.4	0.578	73.0	83.8	82.7	76.1
EMCI		Total scores	77.2	0.551	74.8	79.6	79.2	76.5
SMC	F	Domain scores	67.0	0.326	56.5	74.6	63.7	70.9
SMC		Total scores	68.8	0.364	58.4	76.3	66.2	72.2
SMC	M	Domain scores	90.4	0.726	66.7	97.5	91.7	90.7
SMC		Total scores	86.4	0.596	59.8	94.4	78.2	89.1

## Discussion

Deposition of A$\beta$ is an early recognized marker of AD, detectable as much as a decade prior to symptom onset. A popular therapeutic strategy focuses on amyloid removal, which if implemented preclinically, could potentially change the trajectory of the disease course. Current means of amyloid recognition are either invasive or expensive. Thus, methods that could predict at an individual level who may be most likely to have elevated brain amyloid using easily obtainable clinical data has the potential to reduce costs and speed enrollment in clinical trials of AD disease modifying agents^51,52^.

This study assessed whether incorporation of domain level scoring from cognitive screening tests into a multi-variable ML model could improve diagnostic classification of individuals with early stage AD above total scores. Screening tests have been shown to insensitive to the earliest cognitive changes in AD and we hypothesized that building a model that could presumably integrate a more granular picture of an individual’s cognition (such as isolated weaknesses in verbal memory) would significantly improve model accuracy over total scores^53,54^. As hypothesized, in most of the subgroups analyzed, incorporation of domain level performance but this was neither robust nor true for each subgroup. In particular, we found no benefit of incorporating domain level performance in the women groups in the two earliest stages of AD (SMC and EMCI). This is not entirely unexpected given that women have known advantages over men in verbal memory^19^ and the screening tests sampled—MoCA and ADAS-Cog—rely heavily on verbal memory tasks. The model’s discriminative accuracy lagged significantly behind that for men in the earliest stages of disease followed by a significant improvement in those women who had been diagnosed with LMCI. Women have been shown to decline more rapidly than men in AD and the improved accuracy of the model to classify women in LMCI as opposed to SMC or EMCI may reflect the accelerated failure of memory networks that may occur later in women compared to men^55^. The low discriminative accuracy of the model in SMC (68.8) and EMCI women (71.3), even with the incorporation of APOE $\varepsilon 4$ status indicates that it is challenging to accurately predict amyloid status in women in early stages of AD.

A novel feature of our approach is the development of a new feature selection method based on the stochastic ordering of features within each subgroup^45,56^. This new feature selection method reduced the computational intensity from exponential to linear, making the search for the optimal set of features computationally feasible. The proposed stochastic ordering seemed to work very well in general. The presence of the APOE $\varepsilon 4$ allele is highly correlated with amyloid positivity and we saw that this was the optimal feature seen in each diagnostic group. It was notable that performance on the delayed memory of the MoCA did not contribute significantly to the model’s ability to predict underlying amyloid status. It is likely that having only 5-items in the delayed memory task is neither sensitive nor specific for predicting amyloid accumulation in early stage AD.

One of the limitations of this study is that the samples utilized in this study are not demographically representative of the general population and thus not fully representative of populations who would participate in community screenings. In addition, it would be unlikely to know the APOE $\varepsilon 4$ status of individuals participating in screening events. Diagnostic confirmation of AD is completed with expensive (amyloid PET) and invasive (CSF for amyloid beta) confirmatory studies and there are limited datasets that would allow us to confirm AD in large enough datasets to confirm diagnostic status. Future work should focus on developing ML algorithms using data collected from community samples to test whether these strategies are adequate to meet the challenge of the affordable and accurate diagnosis of individuals with early stage AD. In addition, the sample sizes in each subgroup are not large enough to conduct a three-fold separation into training^30,33^, testing, and validation data sets, as suggested by one of the reviewers. We consider this is an attractive approach to overcome the challenge of identifying an independent data set from another study as the validation data. Due to the lack of a validation data set, the presented performance matrix may be lower as the variations of data sets.

Current approaches to early identification of AD still rely on cost prohibitive, labor-intensive, and expensive diagnostic tests^57^. These diagnostic tests are typically only available in a limited number of tertiary care centers^58^. Furthermore, many psychometric tests used to support a diagnosis are available in only a limited number of languages and may not have options for hearing or visually impaired individuals^59^. This often means an AD diagnosis can be missed or delayed for years^60^. The impact is even more dramatic on the AD drug development pipeline which has seen significant bottlenecks in recruitment for early stage trials and studies composed of largely homogeneous populations^61,62^.

We proposed a new feature selection method based on the stochastic ordering of features within each subgroup. This new feature selection method reduces the computational intensity from exponential to linear, which makes the search for the optimal set of features computationally feasible^10,56,63,64^. The proposed stochastic ordering works very well in general. We did notice the issue of the optimal ML model for the SMC male group where the optimal model was achieved when all the features were included in the model. This was partially caused by the method to determine the feature ordering. For simplicity, the binary logistic regression with the AIC criteria was used for the stochastic ordering. For that subgroup (the SMC male), the final optimal ML method is a tree based method which could be very different from a logistic regression. We would consider this as future work to identity simple statistical models for feature ordering for each ML method.

Patients could be pre-screened with these tools and those that are most likely to have brain amyloid would undergo confirmatory testing with PET amyloid imaging or CSF studies. In conjunction with identifying those at a high risk of amyloid/tau pathology, we hypothesize that ML approaches will be able to estimate the likelihood of disease progression over a defined period. Enrolling patients with a high likelihood of progression will help reduce the chance of a failed trial due to lack of decline in the placebo group. AD and other neurodegenerative disorders cause characteristic patterns of cognitive decline that can be captured by neuropsychological assessments (e.g., the Alzheimer’s Disease Assessment Scale-Cognitive Subscale (ADAS-Cog) or a Neuropsychological Test battery (NTB)). Using novel ML methods based on newly discovered risk factors and biomarkers (e.g., stroke, diabetes, and basal forebrain volume^65,66^) for cognitive decline, our research will increase the understanding of how newly discovered risk factors and biomarkers contribute to prediction of AD biomarkers.

As predicted, the present study showed that for women with no measurable memory deficits, only three cognitive test features, only one related to memory, were included in the optimal model for predicting presence of brain beta amyloid in women with SMC. In contrast, the model for SMC men included all input features. For women with EMCI, no cognitive tests were included as input features in the optimal model, with only age and APOE $\varepsilon 4$ status as most useful in prediction of brain amyloid beta. The model for EMCI men featured only three cognitive test features, including delayed recall memory and object naming, deficits in which are typically thought of as hallmarks of early AD. This pattern again suggests that cognitive assessments may not predict presence of AD pathology in women as effectively as they do in men at early disease stages. In contrast, at the LMCI stage, a broader range of cognitive test scores were included as features in optimal model for women than the one for men. Recall and recognition memory scores were among those included. This finding is consistent with studies showing that women with brain beta amyloid decline cognitively more quickly than men—therefore, cognitive test scores would be expected to better differentiate amyloid positive vs. negative women than men.

The current approach differs from many machine learning analyses, which attempt to predict future cognitive decline using current biomarker status^67–69^. Our analysis adds uniquely to the literature by showing that current cognitive status can accurately predict current amyloid status, particularly in men with SMC. This knowledge could be applied to improve the odds that clinical trials and research studies without access to amyloid PET imaging are including amyloid positive SMC men in their cohorts (i.e., those with preclinical AD), and excluding those with non-AD SMC. This would increase the power of such studies to find results relevant to early disease process in preclinical AD men. Unfortunately, our finding is also consistent with our and others’ prior work showing cognitive tests may not be sufficient to identify women with preclinical AD^27,70^. Practically, this means that without biomarker confirmation or more comprehensive cognitive assessment, women included in SMC groups in clinical trials and research may be more heterogeneous than SMC men. Such heterogeneity could lead to lack of effects or could underlie some findings of sex differences in Alzheimer’s disease.

## Data Availability

Data used in preparation of this article were obtained from the Alzheimer’s disease Neuroimaging Initiative (ADNI) database (http://adni.loni.usc.edu). Thus, the investigators within the ADNI contributed to the design and implementation of ADNI and/or provided data, but did not participate in this analysis or the writing of this report. A complete listing of ADNI investigators can be found at its website.
